# 肺癌的筛查——机遇与挑战

**DOI:** 10.3779/j.issn.1009-3419.2015.12.01

**Published:** 2015-12-20

**Authors:** 立功 聂

**Affiliations:** 100034 北京，北京大学第一医院呼吸内科 Department of Respiratory Medicine, Peking University First Hospital, Beijing 100034, China

肺癌的发病率和死亡率在实体瘤中均高居榜首，在2008年，估计全世界肺癌新发病例为160万，占所有恶性肿瘤的12.7%，死于肺癌的病例数为140万，占死于所有恶性肿瘤的18.2%^[1]^。在我国也是如此，40年前，肺癌是我国恶性肿瘤的第5位死因，而如今，我国肺癌在全部恶性肿瘤的发病和死亡中均处于首位，以北京为例，1974年-2003年间肺癌的死亡人数增加了250%^[2]^。肺癌的发病率居高不下主要是与吸烟人群不断增多有关，在美国，85%的肺癌是由于吸烟所致^[3]^，在我国，除了吸烟因素以外，越来越严重的空气污染也功不可没。肺癌除了发病率高以外，治疗效果也不尽如人意，尽管现在的治疗取得了长足的进步，但是5年存活率不足18%^[4, 5]^，究其原因，主要是不能早期发现和诊断，有症状的患者就诊时75%已经是局部晚期或已有转移^[3]^，而早期肺癌有较好的5年存活率，I期肺癌的5年存活率可达60%-80%^[6, 7]^。因此，许多年来，各国学者在如何发现早期肺癌方面做了大量的研究，包括痰中找瘤细胞、胸部X线检查以及应用低剂量计算机断层扫描（low-dose computed tomography, LDCT）进行筛查等，近来发现应用LDCT筛查可以降低肺癌死亡率^[8]^，在此，我们就LDCT筛查肺癌所带来的收益和风险进行综述。

## 1 LDCT筛查的收益

在20世纪50年代至80年代，各国学者试图通过痰找瘤细胞、胸部X线筛查肺癌，结果是通过胸部X线可以发现更多的早期肺癌，但是不能降低肺癌的死亡率。随着医学影像学的发展，通过胸部CT检查可以发现早期肺癌，因此，21世纪初许多学者试图通过LDCT筛查肺癌，以期降低肺癌的死亡率。美国肺癌筛查研究组在2002年8月-2004年4月应用LDCT筛查肺癌，即国家肺癌筛查试验（The National Lung Screening Trial, NLST）^[8]^，入组标准是年龄在55岁-74岁、吸烟至少30包年、戒烟时间少于15年，同时在18个月内未进行过胸部CT检查、未诊断过肺癌、无咯血、无一年内不明原因的体重下降超过6.8 kg。共入组53, 454例，随机分为LDCT组和胸部X线组，进行3次LDCT检查，分别是入组时的基线检查以及第一年末和第二年末的检查。结果显示与对照组相比，LDCT组肺癌死亡率下降了20%，全因死亡率下降了6.7%；同时显示入组者的戒烟成功率也有提高^[4, 9]^。

与此同时，欧洲也进行了类似的临床研究^[10, 11]^，只是入组标准稍有不同，但是并没有出现我们期待的阳性结果，其原因我们后面将详细讨论。

美国预防服务工作组（U.S. Preventive Services Task Force, USPSTF）根据NLST的结果，发布了新的肺癌筛查指南^[12]^，对筛查人群和方法稍作修改，年龄为55岁-80岁，每年进行LDCT检查，其他方面未做修改。

## 2 筛查带来的问题

正如NLST作者所述^[8]^，肺癌的筛查还面临许多问题，如筛查人群的选择、假阳性结节的后续诊断所带来的副作用、过度诊断（overdiagnosis）以及辐射的问题，我们将逐一讨论。

### 2.1 筛查人群的选择

如前所述，在NLST中，与对照组比较，进行LDCT检查组的肺癌病死率和全因死亡率分别下降20%和6.7%^[8]^，而在欧洲的研究中^[10, 11]^两组间并没有差别。现在我们合并分析这些研究发现，除了样本量有差别外，在入组标准上有些区别，主要体现在吸烟程度上，在NLST中是 > 30包年，而在欧洲的研究中是 > 20包年；另外较NLST人群年轻，前者人群患肺癌的可能性更大，是更高危的人群，因此学者们认为对于高危人群进行LDCT筛查，可以降低肺癌病死率，而对低危人群并没有此作用^[3]^。在高危人群中，挽救1例肺癌患者的生命需要筛查82人，而在低危人群需要筛查3, 180人。

如何定义高危人群，也就是说什么样的人适合进行LDCT筛查，吸烟的程度只是其中的一个参考因素，应该还有其他一些临床因素可供我们参考。Sanchez-Salcedo等^[13]^应用NLST入组标准观察了一个西班牙和美国的肺癌筛查研究的人群，结果发现分别只有36%和59%的人群符合NLST标准，按此标准每年进行筛查，就可能有39%的肺癌没有被发现，如果对符合NLST标准或有肺气肿者进行每年筛查，尽管筛查的人数减少了52%，但是能发现更多的肺癌（西班牙研究中88%、美国研究中95%的肺癌可以被发现），因此，二者结合选择筛查人群，可以发现更多的肺癌，并且漏诊较少。也有学者发现对不吸烟者而言，如果出现肺气肿也预示着肺癌的发病率较高，Henschke等^[14]^报道，正在吸烟者和曾经吸烟者，如果出现肺气肿，肺癌的发病率会翻番，分别是2.3% *vs* 1.1%和1.8% *vs* 0.9%；而不吸烟出现肺气肿者的肺癌发病率增加约6倍，2.6% *vs* 0.4%，这也提示我们对于不吸烟而有肺气肿者是否应该纳入筛查，值得今后研究。

### 2.2 假阳性的问题

在进行筛查时的基线检查中，有27%会出现阳性结果，但96%为假阳性^[15]^。NLST中^[8]^，在实验过程中，阳性发现在LDCT组和胸部X线组分别有39.1%和16.0%，而假阳性率分别是96.4%和94.5%，对这些假阳性需要进行后续的CT、正电子发射型计算机断层显像（positron emission computed tomography, PET）和有创检查予以证实。在NLST后续检查中，每进行245次LDCT和81次胸部X线检查就会出现一次并发症；NLST中1.2%的患者没有发现肺癌而进行了有创检查，如针吸活检或支气管镜检查，0.7%的患者没有肺癌而进行了胸腔镜、纵隔镜和开胸检查；对于良性结节进行有创检查也较常见，NLST中达到了73%；有创检查后60天内死亡人数分别是LDCT组16例（10例为肺癌），胸部X线组10例（全部为肺癌）^[3, 8, 9, 12]^，这些结果强烈警示我们应该进行更深入的研究以提高诊断的特异性。

### 2.3 过度诊断

过度诊断的定义通常是指在筛查中发现恶性肿瘤，但临床上并不显现；是筛查的固有特性，只存在于试图发现无症状的隐匿^[4, 16]^。过度诊断会带来一系列的后果，需要进行后续的诊断检查，甚至外科手术治疗，而这些检查和治疗并不是必须的，这就给患者带来了心理、生理和经济上的损害，同时患者还要面临有创检查和手术所带来的风险。NLST的研究者们^[8]^当时估计过度诊断率不会太大，但是后续的研究发现在NLST研究中^[16]^，在LDCT组发现的肺癌中多达18%为惰性，为了预防1例肺癌死亡需要筛查320人，同时过度诊断是1.38。Veronesi等^[17]^发现在筛查中大约有25%的恶性肿瘤为生长缓慢或惰性，其中许多是过度诊断。

### 2.4 辐射伤害

医疗检查所带来的辐射损害往往被忽视，随着CT的普及，医疗辐射损害应该引起我们的重视。肺部每次LDCT检查的辐射量大约为2 mSv，常规CT检查的辐射量大约为7 mSv^[18, 19]^，而PET-CT的辐射量达到14 mSv^[9]^。McCunney等^[5]^研究了辐射的损害，按筛查20年-30年计，每年进行一次LDCT，如果每两年发现一个阳性结节，那么该人在此期间所受的辐射量将达到280 mSv-420 mSv，在美国如果50%的目前和曾经吸烟者参与每年LDCT筛查，筛查项目本身会引起36, 000例恶性肿瘤；也有机构估计CT扫描引起的肺癌每年达到4, 100例。按照NLST的资料，估计每筛查2, 500人会有1人死于因辐射导致的恶性肿瘤^[9]^。

## 3 今后的研究方向

由于前述肺癌筛查中还有许多大家担忧的问题，因此，我们必须继续在筛查人群的细化、提高肺癌诊断的特异性、减少过度诊断等方面进行研究。

### 3.1 筛查人群的细化

如前所述，NLST可以降低肺癌病死率，而欧洲的筛查并没有出现同样的结果，究其原因可能是筛查人群的不同所致，但是单纯由吸烟程度和年龄来界定高危人群还有其局限性，如每减少1例肺癌死亡需要筛查320人。Kovalchik等^[20]^对NLST资料进行了详细分析，根据年龄、体重指数、肺癌家族史、吸烟量、戒烟时间以及肺气肿等分为高危和低危人群，结果发现，其中60%的高危人群肺癌死亡占所有筛查人群肺癌死亡的88%，只占所有假阳性率的64%，20%的低危人群肺癌的死亡只占所有筛查人群肺癌死亡的1%。Tammemagi等^[21]^应用自行建立的模型验证NLST的结果，发现与NLST入组标准比较，应用PLCO_m2012_标准可以提高筛查敏感性（83.0% *vs* 71.1%）和阳性预测值（4.0% *vs* 3.4%），因此，应用PLCO_m2012_模型在发现肺癌上较NLST标准更为敏感。因此，如果我们能够很好的界定筛查人群的标准，不但能提高敏感性，还可以减少了筛查人数。

### 3.2 提高肺癌诊断的特异性

在NLST^[8]^中，LDCT组和胸部X线组分别有39.1%和16.0%参与者至少出现一次阳性结果，而两组的假阳性率分别达到96.4%和94.5%，这也表明后续需要做许多工作来证实诊断，如果在发现阳性结节的同时，加入其他参数协助诊断，会对筛查帮助甚大。McWilliams等^[22]^应用基于患者及结节特点的预测模型协助诊断，对结节诊断的准确性有较大帮助，并且对10 mm或更小的结节判断的准确性也达到90%。Mehta等^[23]^在患者临床特点和结节特性的基础上，参考结节的体积，能帮助临床工作者有效判断结节的性质。

如果能在血液中有敏感的标记物帮助判断肺部结节，对临床工作是非常方便的。Bianchi等^[6]^检测高危人群中无症状的早期非小细胞肺癌（non-small cell lung cancer, NSCLC）患者血清中34种miRNA，其准确性达到80%，对无论有无症状的NSCLC患者，均有较好的稳定性和准确性，可以判断良恶性病变。Sozzi等^[24]^研究更能说明问题，他们对参与MILD实验者留取的血浆标本进行检测24种miRNA组成信号分类（miRNA signature classifier, MSC），根据其表达分为高危、中危和低危三组，对肺癌诊断的敏感性和特异性分别是87%和81%，对诊断的阴性预测值为99%，对死亡的预测值达到99.86%；在LDCT组的假阳性率为19.4%，加入MSC后，假阳性率下降到3.7%，下降了5倍，因此在筛查的同时参考MSC的结果明显降低假阳性率，值得今后进行前瞻性研究。

### 3.3 过度诊断

目前没有更好的办法准确判断过度诊断，Veronesi等^[17]^应用肿瘤的倍增时间将其分为快速生长（倍增时间少于400 d）、缓慢生长（400 d-599 d）和惰性生长（大于或等于600 d），这种方法比较消极。Sozzi等^[24]^发现MSC与肺癌的预后有关，如果肺癌患者血浆中MSC为低表达，2年存活率为100%，诸如这些标记物能否用来判断惰性肺癌有待今后的研究。

## 4 结束语

肺癌的发病率在不断上升，我国更是明显。尽管晚期肺癌的治疗取得了长足的进步，但是仍不尽如人意，在目前进行源头的控制是行之有效的，包括控烟和筛查，研究已经证明筛查可以降低肺癌的死亡率，但是还有许多挑战等待我们。
